# Systematic reviews and other types of synthesis: commentary on the methodological series published in Epidemiology and Health Services Epidemiologia e Serviços de Saúde

**DOI:** 10.1590/S2237-96222022000300023

**Published:** 2022-12-19

**Authors:** Taís Freire Galvão, Maurício Gomes Pereira

**Affiliations:** 1 Universidade Estadual de Campinas, Faculdade de Ciências Farmacêuticas, Campinas, SP, Brazil; 2 Universidade de Brasília, Faculdade de Medicina, Brasília, DF, Brazil

Previously, Epidemiology and Health Services: Journal of the Brazilian National Health System (*Epidemiologia e Serviços de Saúde: revista do Sistema Único de Saúde* - RESS) published a methodological series on the subject. The six articles that comprised it addressed, in a simplified way, how to prepare and publish systematic reviews of the literature. Each of these articles coped with different steps in the conduct of this type of research: research question;^1^ search for and selection of articles;^2^ data extraction, methodological quality assessment and synthesis of results;^3^ heterogeneity and publication bias;^4^ evaluation of the quality of the evidence generated;^5^ and drafting, publication of the results and critical appraisal of the review.^6^ At the end of the series, the Portuguese translation of the guide for writing systematic reviews was also published, the Preferred Reporting Items for Systematic Reviews and Meta-Analyses (PRISMA), which now has a translation of the PRISMA 2020 update.^7^ This material - the series and guide - provides readers with the rationale and indication of tools for conducting systematic reviews.

In the occasion of the 30^th^ anniversary of RESS, the series was revised in order to complement it. At this special moment, we celebrate the strength of the journal, its three decades of growth and consolidation and, together with our readers, we propose to contribute to the journal through this reflection on the area. It is worth highlighting that the focus of this commentary is not only the methodological approach, subject of the series and writing guide aforementioned, which we recommend to the reader who is eager to learn more about how to conduct this type of research,^1^ ^-^ ^7^ but also to comment on what has already been put forward.

For those who follow publications of literature reviews in scientific journals, a first observation lies in its rapid methodological development. Innovative approaches to its conduct have been proposed, often accompanied by new terms, which may confuse readers. Initially, an introduction to the article compares the systematic review with the traditional one. Then, other types of evidence synthesis are addressed and suggestions on how to learn more about the subject are presented.

## SYSTEMATIC REVIEW AND NARRATIVE REVIEW

Literature reviews are very useful because they allow you to quickly get up to date on a topic by accessing and consulting this type of publication; in addition, they propose actions, recommendations and new research. Every research project or article provides information about studies conducted, which are able to help to understand and justify the research theme of the project or article, usually presented in the introduction of these documents. Thus, it is consistent with this reality to state that every research professional conducts a literature review in some way.

Two types of review predominate: systematics and narrative (Box 1). When preparing a systematic review, its author starts from the researcher’s perspective. It aims to objectively search the literature, escaping subjectivity of personal opinion. Therefore, a guiding research question is formulated, to be answered using a standardized and transparent method, in order to identify and summarize relevant studies. Among the precautions inherent to this process, it is important not to incur bias in the selection of articles for consultation and analysis. Moreover, there is a possibility that systematic review expresses a fragmented view of the situation, depending on the formulated question and on the existing research on the subject. Finally, when well conducted, the systematic review should answer, objectively and clearly, the specific question that motivated its conduction.

**Box 1:** - Main characteristics of systematic and narrative reviews

Type	Objective	Question	Limitations	Example
Systematic	To answer in specificity to the outlined question, with an objective answer to the outcome.	Structured using PICOT.^a^	Long lead time; requires adequate staff and resources; methods can be distorted.	Prevalence of depression morbidity among Brazilian adults: a systematic review and meta-analysis DOI: 10.1590/1516-4446-2013-1294
Narrative	To enable first contact or update knowledge on a topic.	Broad and unstructured.	Influenced by the author’s knowledge and experience.	*Aedes aegypti* control strategies: a review DOI: 10.5123/S1679-49742016000200017

a) PICOT: Population, intervention or exposure, comparison, outcome, type of study.

Note: Frequency reviews follow the “population, outcome and type of study” (POT) structure; in these cases, the effect of an intervention or exposure is not important, but rather the disease frequency.

The review that provides comprehensive information on a topic, addresses the characteristics of a disease, its causes, most affected groups, treatment, prevention, etc. is called narrative review. Chapters of books and articles in which a subject specialist discusses a subject and summarizes the literature related to it, according to his or her own conception, are also called narrative reviews, and they are widely used. Specific questions, such as those formulated in systematic reviews, are also found in narrative reviews. The non-systematic choice of articles for inclusion in the narrative review, however, does not ensure that the answer provided is consistent with the totality of available evidence. A text with such characteristics is influenced by the author’s experience and by his convictions and opinions, whether updated or not. However, the narrative review has been very useful for the first contact with a theme or the update on questions about a subject that is already known.

The fact that the systematic review employs a standardized and transparent method does not exempt it from failure. Each of the steps can be well or poorly conducted, depending on how the procedures were performed. The quality of the evidence generated depends on the primary studies. Regardless of the effort of the authors, a good systematic review can provide low-quality evidence. Because they have high scientific prestige, systematic reviews can serve commercial interests and thus influence decisions, often misleading the reader who believes they have the best evidence.^8^

The systematic review clearly answers the question that has guided it. At its end, the reader should be explicitly informed about the answer, such as whether or not the therapy works, whether or not the factor is a risk factor or about the prevalence of the disease. A review that performed the recommended steps and shows as results the type of journal, language or impact factor of the publications, rather than the answer on the health outcome, would be a review or bibliometric survey.

## OTHER TYPES OF LITERATURE REVIEWS

Other types of reviews start from the question and specification of the methods used to synthesize the evidence. In addition to the authors’ knowledge, they are based on interactive guide to types of reviews of Temple University Libraries (https://guides.temple.edu/systematicreviews), which is recommended to consult. Their main characteristics are summarized in Box 2.

**Box 2:** - Types an characteristics of other literature reviews

Type	Objective	Question	Limitations	Example
Rapid	To answer a clinical or management question quickly.	Specific and structured in PICO format.^a^	Subject to bias, especially selection bias.	Digital contact tracing technologies in epidemics: a rapid review DOI: 10.1002/14651858.CD013699
Scoping	To know the availability and focus of the research.	Broad and topic-based.	Subject to inconsistency, given the multiple possibilities of methods and types of synthesis.	Approaches for defining and assessing nursing informatics competencies: a scoping review DOI: 10.11124/JBIES-20-00100
Mapping	To provide visual synthesis of data and identify evidence gaps.	Broad, but question-based and not only topic-based, can follow the PICO structure.^a^	Subject to inconsistency, requires experience and resources for visual synthesis.	An evidence map of research linking dietary sugars to potentially related health outcomes DOI: 10.1093/cdn/nzy059
Integrative	To integrate empirical and theoretical evidence for overall understanding of a problem.	Variable, problem-based, or practice-based.	Poorly standardized methods; combining different research may affect the rigor and accuracy of the data.	Advance care planning for adults with CKD: a systematic integrative review DOI: 10.1053/j.ajkd.2013.12.007
Overview	To combine different available reviews for better clinical decision.	Specific and structured in PICO format, and may include different interventions or population groups.	Subject to inappropriate comparisons on effects of interventions and overlapping primary studies.	Endometriosis: an overview of Cochrane Reviews DOI: 10.1002/14651858.CD009590.pub2

a) PICO: Population, intervention or exposure, comparison and outcome.

The rapid review follows the same steps as a systematic review; however, it restricts procedures to be completed in a short time, usually up to three months.^9^ Its search may be restricted to one or two bibliographic databases, only. The remaining steps, done in duplicate in the systematic review, can be performed by a single researcher in the rapid review.^9^

Scoping and mapping reviews aim to define what research exists in the area, in order to identify knowledge gaps that need to be addressed by further research. Such reviews start from questions that are broader than those of systematic reviews, and they are not focused on research results, but rather on the existence of research and knowledge gaps in the topic under consideration. One characteristic that distinguishes the mapping review is the fact that it is focused on the composition of a visual synthesis, therefore its questions are usually less broad than those of the scope review.

The integrative review brings together research of a distinct methodological nature, for a more comprehensive understanding of a health phenomenon. Widely used in the nursing area, this review can include qualitative and quantitative preclinical and clinical research in its eligible studies. Through the compatibility of information of diverse nature, it aims to present the synthesis of different types of knowledge on the subject. Furthermore, it is worth clarifying how the integrative review was conducted. Given the combination of methods used for conducting it, it would correspond to a type of narrative review. Depending on the classification system, these reviews are not part of the group of types of evidence synthesis.^10^

Reviews that summarize other literature reviews on a common theme are called overview of reviews. The advantage of this type of synthesis is the possibility of grouping different questions in the structure “Population, Intervention, Comparison and Outcome” (PICO), in order to provide a broader view of the subject. Another positive aspect is the rigor when conducting it, based on systematic reviews and following a procedure similar to the preparation of this type of review.

## ADDITIONAL RESOURCES

In order to conclude this contribution to the debate, institutions and material for methodological support for the preparation of systematic reviews are presented in the Supplementary Material to this article. It is noteworthy that in addition to the points highlighted in this material, these institutions offer courses, seminars and scientific events for methodological improvement in the area. It is worth noting that other relevant institutions are not included in this list.

Free courses on preparation methods or simply to improve the management of this type of research, an increasingly necessary skill for researchers and health professionals, are also available. Among these courses “Evidence-Based Health” (https://www.coursera.org/learn/sbe) and “Systematic Review and Meta-Analysis” (https://www.coursera.org/learn/revisao-sistematica) stand out. They are offered on the Coursera platform, coordinated by the author and a team of experienced researchers. In addition to offering the basic procedures for critical appraisal of scientific literature and preparation of systematic reviews, the courses indicate additional sources and resources for deepening the theme.

## Supplementary Material - Main institutions that provide methodological support for the preparation of systematic reviews

**Table:**

Institution	Description	Main resource available	Website
Cochrane	Leading organization in the preparation of systematic reviews on health interventions.	Handbook for conducting systematic reviews.	https://www.cochrane.org/
Joanna Briggs Institute	Organização que promove evidências de implementação em serviços de saúde.	Tools for critical appraisal of primary studies.	https://jbi.global/
Campbell Collaboration	Research network that generates evidence synthesis on social sciences.	Repository for collaboration reviews.	https://www.campbellcollaboration.org
Systematic Review Center for Laboratory Animal Experimentation (SYRCLE)	Center for dissemination of methodology of systematic reviews of animal studies.	Tool for critical appraisal of animal studies.	https://www.syrcle.network/ **DOI:** 10.1186/1471-2288-14-43
